# Intratumoral delivery of IL-18 naked DNA induces T-cell activation and Th1 response in a mouse hepatic cancer model

**DOI:** 10.1186/1471-2407-7-87

**Published:** 2007-05-23

**Authors:** Chi-Young Chang, Jienny Lee, Eun-Young Kim, Hae-Jung Park, Choon-Hyuck Kwon, Jae-Won Joh, Sung-Joo Kim

**Affiliations:** 1 Transplantation Research Center, Samsung Medical Center, Sungkyunkwan University School of Medicine, 50 Ilwon-Dong, Kangnam-Ku, Seoul 135-710, Republic of Korea; 2 Department of Surgery, Samsung Medical Center, Sungkyunkwan University School of Medicine, 50 Ilwon-Dong, Kangnam-Ku, Seoul 135-710, Republic of Korea

## Abstract

**Background:**

The novel cytokine, interleukin (IL)-18, is a strong interferon-γ inducer and costimulatory factor in Th1 cell activation. IL-18 triggers IFN-γ production and enhances cytolytic activity in both T and NK cells. However, the exact mechanism of antitumor action of IL-18 remains to be clarified. To determine the effects of IL-18 plasmid DNA on hepatic cancer in mice, CT26 murine colon adenocarcinoma cells were established in mouse liver.

**Methods:**

Plasmid vectors encoding IL-18 were transferred directly into the liver 7 days after tumor injection to restrict IL-18 expression within the tumor site. The IL-18 protein level was increased in the liver 4 days after plasmid injection, and a marked antitumoral effect was observed at day 7. Antitumor effects were evaluated by measuring tumor regression, immune cell population, and IFN-γ production.

**Results:**

The IL-18 plasmid controlled the growth of hepatic tumors and proliferation of splenic immune cells. Moreover, treatment of CT26 tumors with the IL-18 plasmid significantly enhanced the population of the effector T and NK cells in the spleen and peripheral blood. In spleen, the population of CD4^+^CD62^Low ^cells was augmented in response to IL-18 on day 7. These results are consistent with the increase in CD4^+ ^T cells secreting IFN-γ, but not CD8^+ ^T cells. The marked reduction of tumor growth in tumor-bearing mice was associated with the maintenance of IFN-γ production in spleen in response to IL-18. These antitumoral effects were maintained until 14 days after plasmid injection.

**Conclusion:**

Our results suggest that direct plasmid DNA transfer of IL-18 with no accompanying reagents to augment transfection efficiency may be useful in tumor immunotherapy.

## Background

Effective eradication of established tumors and generation of a lasting systemic immune response with a simple gene delivery system are important goals for cancer gene immunotherapy [1]. Cytokines are the most extensively studied immunostimulatory agents in cancer gene therapy [2]. Interferon-γ-inducing factor (IL-18) is a recently characterized murine and human cytokine. The murine IL-18 gene encodes a precursor protein of 192 amino acids, which is processed to a mature protein containing 157 residues [3]. This cytokine, produced by Kupffer cells, is a potent inducer of IFN-γ production by T cells and a costimulatory factor for T cell activation [4,5]. Accumulating evidence that IL-18 is a multifunctional cytokine that shares several biological activities with IL-12 has led to a series of studies on its effects on T and NK cells [4]. Analogous to IL-12, IL-18 stimulates T cell proliferation and NK cell activity [3]. The finding that IL-18 stimulates the differentiation of Th1 cells that produce cytokines necessary for the development of cell-mediated immune responses suggests a prominent role in defense against tumors [6]. Similarly, high IFN-γ levels are induced by IL-18 in splenic-derived CD4 T cells in the presence of B cells or adherent cells [7]. Furthermore, IL-18-transfected tumor cell vaccines and local delivery of the IL-18 gene as naked DNA via a gene gun or viral vectors has been extensively investigated. Numerous animal studies show that IL-18 has potent antitumor effects, but induces side-effects upon systemic administration [8]. Direct intratumoral DNA administration is reliable and reproducible, and may limit the need for systemic cytokine administration [9-11]. Here, we report the effects of direct intratumoral injection of a nonviral plasmid vector encoding murine IL-18 DNA in established CT26 liver tumors [12].

## Methods

### Animals and cell lines

BALB/c (H-2^d^) mice (6 to 8 weeks old) were obtained from the Jackson Laboratory (Bar Harbor, ME, USA). All mice were housed in specific pathogen-free conditions, in accordance with institutional guidelines. CT26 tumor cells were maintained in RPMI 1640 cell culture medium (Biowhittaker, Walkersville, MD, USA) supplemented with 10% heat-inactivated fetal bovine serum, 2 mM L-glutamine, 100 U/ml streptomycin, and 100 μg/ml penicillin.

### Plasmids

The 11 kb mIL-18 DNA expression plasmid vector, pCEP4-mIL18, was constructed using a CMV early enhancer/promoter/EBNA-1-based pCEP4 plasmid vector (Invitrogen, San Diego, CA) with an ampicillin selection gene. Plasmid DNA was purified in the absence of ethidium bromide using a commercial column chromatography method, according to the manufacturer's protocol (Qiagen, Chatsworth, CA).

### Tumor model and therapeutic protocol

CT26, an undifferentiated murine adenocarcinoma, was induced by rectal injection of *N*-nitroso-*N*-methylurethane in BALB/c mice. Colorectal cancer is often metastatic, the most common site of metastasis being the liver. CT26 cells were suspended for implantation at 1 × 10^5 ^cells/50 μl saline. Colon carcinoma was established in the left lateral lobe of 6- to 8-week-old male BALB/c mice by an abdominal operation. Treatments began 7 days after a defined solid tumor growth was identified within the injected lobe. For intratumoral injection, naked DNA (10, 25, and 50 μg) was diluted in 50 μl saline, and injected into the parenchyma of the lower surface of the left liver lobe via insulin syringes (31 gauge, 0.8 inch needles; Becton Dickinson, Franklin Lake, NJ). Mice were sacrificed at 1, 4, 7, and 14 days after DNA treatment, and tumor growth monitored by measuring liver weight [13].

### Flow cytometric analysis

Total nuclear cells in the peripheral blood were isolated by erythrocyte lysis with ammonium chloride solution (PharM Lyse, Becton Dickinson). Single spleen cell suspensions were obtained by teasing apart spleen tissue and disaggregating cells through a 70 μm mesh. Briefly, splenocytes and PBMCs were incubated with PE-, Cy5, or FITC-conjugated Abs, and the corresponding isotypes (purchased from Pharmingen, San Diego, CA, USA). Anti-mouse NK (clone DX5), -CD3, -CD4, -CD8, -Cd62L, -CD69, -CD19, and -CD11b Abs were used to stain the populations of NK cells, macrophages, T-cells, and B-cells in the spleen. Samples were analyzed for surface phenotypes using FACS Vantage (Becton-Dickinson, San Jose, CA, USA) and CellQuest software (Becton-Dickinson Labware).

### Flow cytometric assessment of intracellular cytokine production

For parallel evaluation of intracellular cytokines, surface staining was initially performed for CT26 lysate-stimulated splenocytes from each group. Cells were fixed with 4% paraformaldehyde solution for 20 min, treated with permeabilization buffer (0.1% saponin, 1% FBS in PBS) for 5 min at RT in the dark, and washed. Intracellular staining was performed by incubation of mAb to IFN-γ with cell pellets for 20 min at RT in the dark. Cells were washed, and measured using a flow cytometer.

### Assessment of cytokines (IL-18, IFN-γ)

We obtained total liver protein by homogenization of frozen tissue in extraction buffer containing 1% Triton X-100, 10 mM Tris-HCL (pH 7.6), 5 mM EDTA, 50 mM NaCl, 30 mM Na_4_P_2_O_7_, 50 mM NaF, 200 μM Na_3_VO_4_, 2 mM PMSF, 5 μg/ml aprotinin, 1 μg/ml pepstatin A, and 2 μg of leupeptin. The suspension was centrifuged at 14,000 r.p.m. for 20 min at 4°, and the supernatant stored at -70°. Supernatant fractions were analyzed with the IL-18 ELISA kit (MBL, Nagoya, Japan). IFN-γ concentrations in the splenocyte culture supernatants were determined with specific ELISA performed according to the manufacturer's instructions (R&D Systems, Minneapolis, MN, USA). The absolute cytokine levels were calculated by comparison to assay performance in the presence of known quantities of recombinant cytokine standards.

Statistical Analysis

An unpaired *t*-test was employed to compare tumor weight, quantitative cytokine production, and flow cytometry analyses. All analyses were performed with SigmaPlot 2000. *P *< 0.05 was considered significant.

## Results

### Levels of transgene expression within the treated tumor site

**Figure 1:**
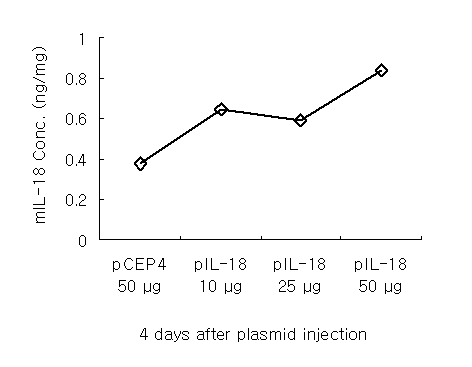
**Detection of mIL-18 plasmid expression in DNA treatment sites**. BALB/c mice were inoculated with murine CT26 cells in the left liver lobe, and treated intratumorally with varying doses of control plasmid or that expressing IL-18 until day 7 after tumor inoculation. Liver tissue was obtained on day 4 after direct injection of IL-18 plasmid DNA, and ELISA used to evaluate IL-18 protein levels. Representative data determined from two separate experiments.

To determine the efficiency of gene transfection, mouse IL-18 protein expression in the tumor site was evaluated by ELISA. mIL-18 protein was detected in all samples injected with control vector or IL-18 plasmid (Figure 1). However, significant differences were evident in the mIL-18 protein levels between the control and the 50 μg plasmid-injected group. Protein levels in CT26 treated with control or mIL-18 plasmid vector ranged from 500 to 900 mg in the injected lobe, since tumors developed differentially after each treatment. The IL-18 protein level was 2–2.5 fold higher than background when mice were sacrificed 4 days after plasmid introduction. The results suggest that mIL-18 DNA is expressed in the tumor site, and releases bioactive IL-18.

### *In vivo *kinetic studies of the immune cell population in spleen

**Figure 2:**
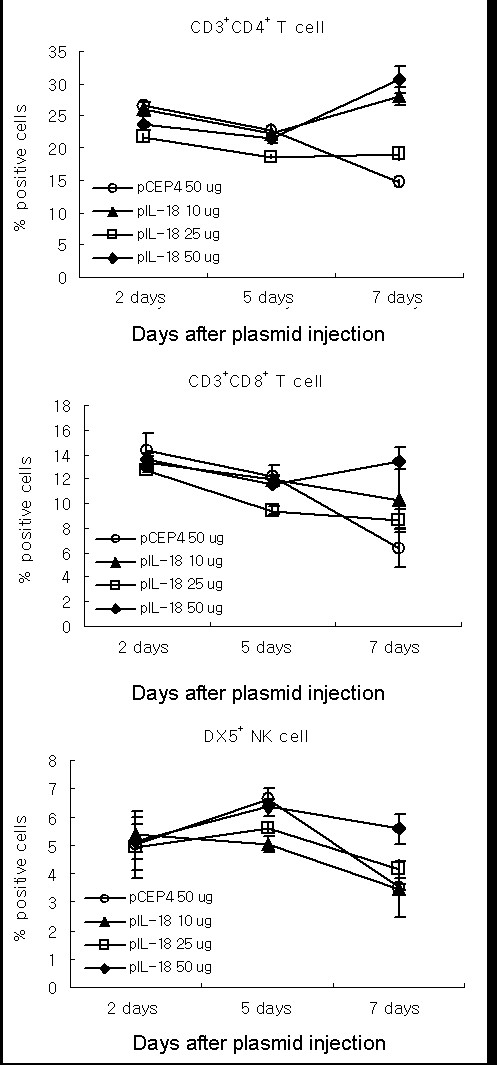
**Immune response is enhanced by a single injection of naked IL-18 plasmid DNA**. Each group (consisting of 4 mice) was administered an intratumoral injection of pCEP4 or naked mIL-18 DNA. Spleens of CT26 tumor-bearing mice treated with plasmids were harvested at 7 days after plasmid injection, and cells analyzed by flow cytometry using the corresponding FITC- or PE- or PE-Cy5-labeled antibodies and isotype control.

We performed phenotype analysis of spleen cells from pCEP4, mIL-18 DNA-treated CT26 tumor-bearing mice on days 2, 5, and 7 after gene treatment, using flow cytometry. The antitumoral response of tumor-bearing mice treated with 50 μg of DNA was significantly greater than that of mice administered 10 or 25 μg DNA. Intratumoral delivery of 50 μg of plasmid DNA for each of four treatments resulted in immune cell augmentation in CD4^+ ^cells (30.6% total lymphocytes versus 15% in control), CD8^+ ^cells (13.5% total lymphocytes versus 6% in control), and NK cells (5.6% total lymphocytes versus 3.5% in control) on day 7 (Figure 2). Other groups report that IL-18 upregulates ICAM-1 expression on monocytes and T cells, presenting further evidence of IL-18-stimulated T cell recruitment [14-17]. These findings, together with the above kinetic data on the immune cell populations, suggest that a ~ 2.5-fold higher concentration of IL-18 modulates the immune cell population (T cells and NK cells) around the time of tumor regression.

### Regression of established CT26 hepatic tumors after direct intratumoral mIL-18 gene transfer

**Figure 3:**
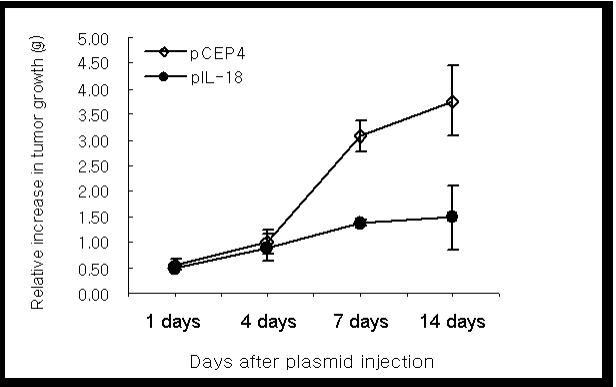
**IL-18 gene transfer results in regression of CT26 liver tumors**. CT26 cells (1.0 × 10^5^) were injected into a left lobe of the livers of 6-8-week-old BALB/c mice. pCEP4 or mIL-18 plasmid DNA was injected directly into the tumors on day 7. Tumor growth was measured on days 1, 4, 7, and 14 after plasmid implantation. Data are presented as the mean of tumor weights of 8 mice/group.

CT26-tumor bearing mice were treated with 50 μg pCEP4 or mIL-18-pCEP4, and tumor growth measured at the indicated times (Figure 3). Tumor growth was significantly lower in the groups treated with mIL-18 plasmid than those administered control vector (pCEP4). Most of the control mice developed multiple large tumors in the liver, but pIL-18 gene therapy markedly inhibited tumor growth at 14 days and 21 days after tumor implantation (see additional file 1). Differences between the control and mIL-18-treated groups were statistically significant (*p *< 0.049 on day 14), as confirmed with the Student's *t *test.

### Regression phenotypic changes of immune effector cells in spleen and blood samples

Changes in cell surface activation markers define specific phases of T cell activation, and may be used to distinguish between naïve, effector, and memory T cell populations [18]. Resting naïve T cells express low levels of CD44 and integrins, LFA-1 and VLA-4, and high levels of CD45RB and CD62L (L-selectin). Upon antigen stimulation, naïve T cells transform into large blastoid cells, and the phenotypes of these effector cells become CD44^hi^LFA-1^hi^VLA-4^hi^CD45RB^lo^CD62L^-^[18,19]. Other activation markers, such as CD25 and CD69, are additionally upregulated [20,21]. Accordingly, flow cytometric analysis of spleen and blood cells from pCEP4 or mIL-18 plasmid-treated CT26 tumor-bearing mice at 7 days after gene treatment was performed. As expected (Table 1), a marked increase (*p *< 0.04) in the CD4^+^CD62^Low ^subset was evident in spleen and blood cells following injection of mIL-18 plasmid DNA (30.05 ± 1.85% in spleen and 30.58 ± 4.59% in blood), compared with the pCEP4 DNA-treated control group (17.75 ± 0.92% in spleen and 17.62 ± 2.15% in blood).

**Table 1:** Frequency of CD4^+^CD62^Low^, CD8^+^CD62^Low^, DX5^+^, CD19^+^cells in spleen and blood: major subsets

Treatment Group	Spleens				Blood			
		
	CD4^+^CD62^Low ^(%)	CD8^+^CD62^Low ^(%)	DX5^+ ^(%)	CD19^+ ^(%)	CD4^+^CD62^Low ^(%)	CD8^+^CD62^Low ^(%)	DX5^+ ^(%)	CD19^+ ^(%)
pCEP4	17.75 ± 0.92	0.997 ± 0.05	3.39 ± 1.26	44.51 ± 3.40	17.62 ± 2.15	5.93 ± 0.96	15.45 ± 1.19	28.97 ± 3.52
pIL-18	30.05 ± 1.85^a^	1.995 ± 0.34	1.73 ± 0.08	41.57 ± 1.09	30.58 ± 4.59^a^	9.88 ± 0.94	18.4 ± 2.91	36.49 ± 5.95

Spleen and blood samples of CT26 tumor-bearing mice treated with pCEP4 or mIL-18 plasmid DNA were harvested 7 days after gene treatment, and cells analyzed by flow cytometry using the corresponding FITC-, PE-, or PE-Cy5-conjugated antibodies and isotype control. Data are presented as means ± SD of 8 mice/group. ^a ^*P *< 0.04, compared with the pCEP4 control-treated group.

**Figure 4:**
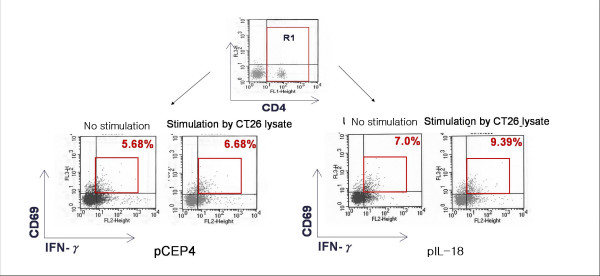
**Intracellular IFN-γ production by CT26-specific T cells from pmIL-18-treated mice**. Splenocytes from CT26-tumor bearing mice treated with three rounds of pCEP4 or mIL-18 were pooled and incubated in medium alone or that containing CT26 lysates. Numbers within the gates in the FACS plots depict the percentage of CD69^+ ^IFN-γ^+ ^CD4^+ ^cells. Data are presented from experiments that were repeated at least twice.

IL-18 induces IFN-γ production in T cells [5,7,22]. To further characterize the mIL-18- induced immune response, intracellular cytokine staining for IFN-γ production was performed for subsets of CT26-specific T cells. Splenocytes were prepared and pooled from CT26 tumor-bearing mice treated with pCEP4 or mIL-18 plasmid incubated with medium alone or supplemented with CT26 lysates. The frequencies of functional CT26-specific T lymphocytes were estimated by intracellular IFN-γ staining, and quantitation of activated CD69^+^, IFN-γ^+^, CD4^+^, and CD8^+ ^cells (Figure 4). While control mice treated with pCEP4 did not generate a CT26-specific T cell response, those administered pmIL-18 contained a significant number of IFN-γ producing CD4^+ ^T cells, but not CD8^+ ^T cells (data not shown). The data suggest that the IL-18-mediated immune response predominantly involves IFN-γ-producing CD4^+ ^T cells.

DNA vaccination of mice resulted in broad immune responses, characterized by the activation of B cells, helper CD4^+ ^and cytotoxic CD8^+ ^T cells [23]. Based on these results, we propose that mIL-18 overexpression in the tumor site induces phenotypic changes in the T cell and B cell subsets, and protects against tumor development by stimulating IFN-γ production by CD4^+ ^T cells.

### Intratumoral injection of mIL-18 plasmid DNA elevates IFN-γ production by splenocytes

**Figure 5:**
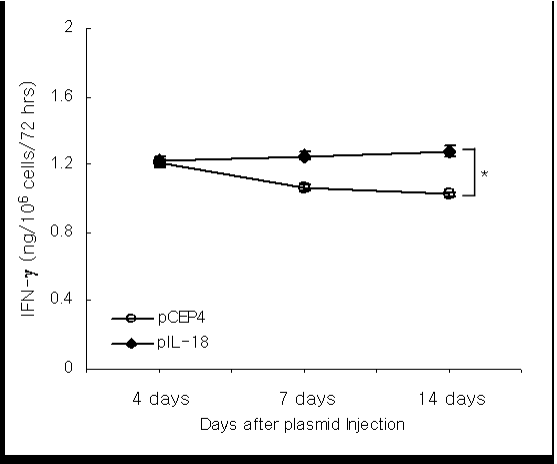
**Elevated IFN-γ levels after intratumoral injection of nonviral mIL-18 plasmid DNA**. Splenocytes from CT26 tumor-bearing mice after gene therapy were collected at 4, 7, and 14 days after gene treatment, and cultured with anti-CD3 and anti-CD28 antibodies for 3 days before performing ELISA (* = 0.006).

The IFN-γ levels of cultured splenocyte supernatants were compared. Enhanced secretion of IFN-γ by cultured splenocytes was evident in animals treated with plasmid mIL-18 DNA (1.278 ± 0.06 ng/10^6 ^cells/72 h), compared to splenocytes from mice treated with pCEP4 control vector (1.026 ± 0.03 ng/10^6 ^cells/72 h; *P *= 0.006). This may explain the continued maintenance of tumor suppression in mIL-18-treated mice, compared to control mice (Figure 5). Interestingly, in our studies with tumor-bearing mice, intratumoral injection with an IL-18 plasmid did not prevent CT26 tumor development, although augmentation of T cells and maintenance of the IFN-γ level during 2 weeks appeared to reject tumors. Thus, while direct cytokine gene transfer possibly has therapeutic potential, expression of cytokine genes may be elevated and sustained significantly during tumor development. Other reports show that the effects of cotransfection of ICE and pro-IL-18 cDNA are superior to those of pro-IL-18 cDNA alone, and result in enhanced bioactivity of IL-18 [24].

## Conclusion

Recent trials using cytokine genes show that intratumoral IL-18 gene transfer is a feasible procedure, but exerts only mild antitumor effects [24]. Gene transfer with increased mIL-18 doses may enhance antitumor efficacy. In this study, we validate the antitumoral efficacy of mIL-18 overexpression in CT26 tumor-bearing mice by using plasmid vectors to transfer the mouse IL-18 gene alone. Our data show marked inhibition of tumor growth and significant phenotypic changes of immune cells, suggesting that mIL-18 intratumoral plasmid transfer may be developed as an effective alternative therapy for cancer treatment with no significant side-effects.

## Competing interests

The author(s) declare that they have no competing interests.

## Authors' contributions

CY carried out the molecular immunology studies, including virus work, and drafted the manuscript. J, HJ and EY performed the *in vivo *study, including mouse surgery, and statistical analyses. CH and JW participated in the design of the study. SJ conceived the study, and aided in its design and coordination. All authors read and approved the final manuscript.

## Pre-publication history

The pre-publication history for this paper can be accessed here:



## Supplementary Material

Additional File 1 Representative results of pIL-18 gene therapy in hepatic tumor bearing mice. Visible tumor foci are generally formed in the liver within a week of injection of CT26 cells, and these grew to a few millimeters in diameter by day 21. Treatment was initiated 7 days after tumor cell injection and representative results of gene therapy were showed at 14 days and 21 days after tumor cell injection. Click here for file
