# Supplementary figures and images for: Intratumoral delivery of IL-18 naked DNA induces T-cell activation and Th1 response in a mouse hepatic cancer model
Source: BMC Cancer. 2007 May 23;7:87. doi: 10.1186/1471-2407-7-87 (PMC1903361; doi:10.1186/1471-2407-7-87)

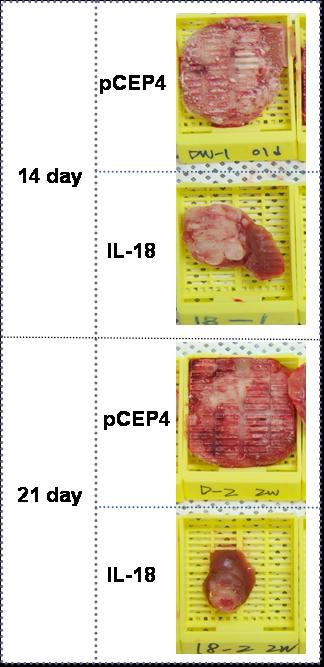

Supplement: Additional File 1 — Representative results of pIL-18 gene therapy in hepatic tumor bearing mice. Visible tumor foci are generally formed in the liver within a week of injection of CT26 cells, and these grew to a few millimeters in diameter by day 21. Treatment was initiated 7 days after tumor cell injection and representative results of gene therapy were showed at 14 days and 21 days after tumor cell injection. [file 1471-2407-7-87-S1.jpeg]
